# Mortality and Clinical Predictors After Percutaneous Mitral Valve Repair for Secondary Mitral Regurgitation: A Systematic Review and Meta-Regression Analysis

**DOI:** 10.3389/fcvm.2022.918712

**Published:** 2022-07-04

**Authors:** Wence Shi, Wenchang Zhang, Da Zhang, Guojie Ye, Chunhua Ding

**Affiliations:** ^1^Aerospace Center Hospital, Beijing, China; ^2^Peking University Aerospace School of Clinical Medicine, Beijing, China

**Keywords:** secondary mitral regurgitation, percutaneous mitral valve repair, atrial fibrillation, left ventricular function, predictor

## Abstract

**Background:**

Percutaneous mitral valve repair (PMVR) provides an available choice for patients suffering from secondary mitral regurgitation (SMR), especially those whose symptoms persist after optimal, conventional, heart-failure therapy. However, conflicting results from clinical trials have created a problem in identifying patients who will benefit the most from PMVR.

**Objective:**

To pool mortality data and assess clinical predictors after PMVR among patients with SMR. To this end, subgroup and meta-regression analyses were additionally performed.

**Methods:**

We searched PubMed, EMBASE, and Cochrane databases, and 13 studies were finally included for meta-analysis. Estimated mortality and 95% confidence intervals (CIs) were obtained using a random-effects proportional meta-analysis. We also carried out a meta-regression analysis to clarify the potential influence of important covariates on mortality.

**Results:**

A total of 1,259 patients with SMR who had undergone PMVR were enrolled in our meta-analysis. The long-term estimated pooled mortality of PMVR was 19.3% (95% CI: 13.6–25.1). Meta-regression analysis showed that mortality was directly proportional to cardiac resynchronization therapy (CRT) (β = 0.009; 95% CI: 0.002–0.016; *p* = 0.009), an effective regurgitant orifice (ERO) (β = 0.009; 95% CI: 0.000–0.018; *p* = 0.047), and a mineralocorticoid receptor antagonist (MRA) use (β = −0.015; 95% CI: −0.023–−0.006; *p* < 0.001). Subgroup analysis indicated that patients with preexisting AF (β = −0.002; 95% CI: −0.005– −0.000; *p* = 0.018) were associated with decreased mortality if they received a mitral annuloplasty device. Among the edge-to-edge repair device group, a higher left ventricular (LV) ejection fraction, or lower LV end-systolic diameter, LV end-systolic volume, and LV end-diastolic volume were proportional to lower mortality.

**Conclusion and Relevance:**

The pooled mortality of PMVR was 19.3% (95% CI: 13.6–25.1). Further meta-regression indicated that AF was associated with a better outcome in conjunction with the use of a mitral annuloplasty device, while better LV functioning predicted a better outcome after the implantation of an edge-to-edge repair device.

## Introduction

Secondary mitral regurgitation (SMR), which is most commonly seen in dilated or ischaemic cardiomyopathies, is associated with significantly poor clinical outcomes and quality of life (1, 2). Although optimal medical therapy may be prescribed, symptoms of heart failure cannot be relieved in certain patients. Surgical mitral valve intervention is still recommended for patients at low surgical risk or for those without advanced left ventricular remodeling (3, 4). However, few therapeutic alternatives have been shown to lower the rate of hospitalization or death in the high-risk group.

The COAPT (Cardiovascular Outcomes Assessment of the MitraClip Percutaneous Therapy for Heart Failure Patients with Functional Mitral Regurgitation) trial (5) and its 3-year follow-up results (6) confirm that percutaneous mitral valve repair (PMVR) is a feasible treatment for moderate-to-severe or severe SMR. Conflicting conclusions from the MITRA-FR (The Percutaneous Repair with the MitraClip Device for Severe Functional/Secondary Mitral Regurgitation) trials (7, 8) render the benefit of PMVR debatable. It appears that a select population fulfilling COAPT inclusion criteria may benefit from PMVR (9, 10).

Long-term follow-up outcomes from the COAPT and MITRA-FR trials, and other recent research, including those providing extra data or using new transcatheter systems, provided us with a tremendous opportunity to pool all the evidence of PMVR for patients with SMR. As it is necessary to recognize patients who might benefit from PMVR the most, a meta-analysis and meta-regression were performed to identify these clinical predictors.

## Methods

We registered our meta-analysis in the International Prospective Register of Systematic Review (CRD42022321423), and Preferred Reporting Items for Systematic Reviews and Meta-Analyses guidelines was used to design our manuscript (Supplementary Material 1).

### Study Selection Criteria

**Figure 1:**
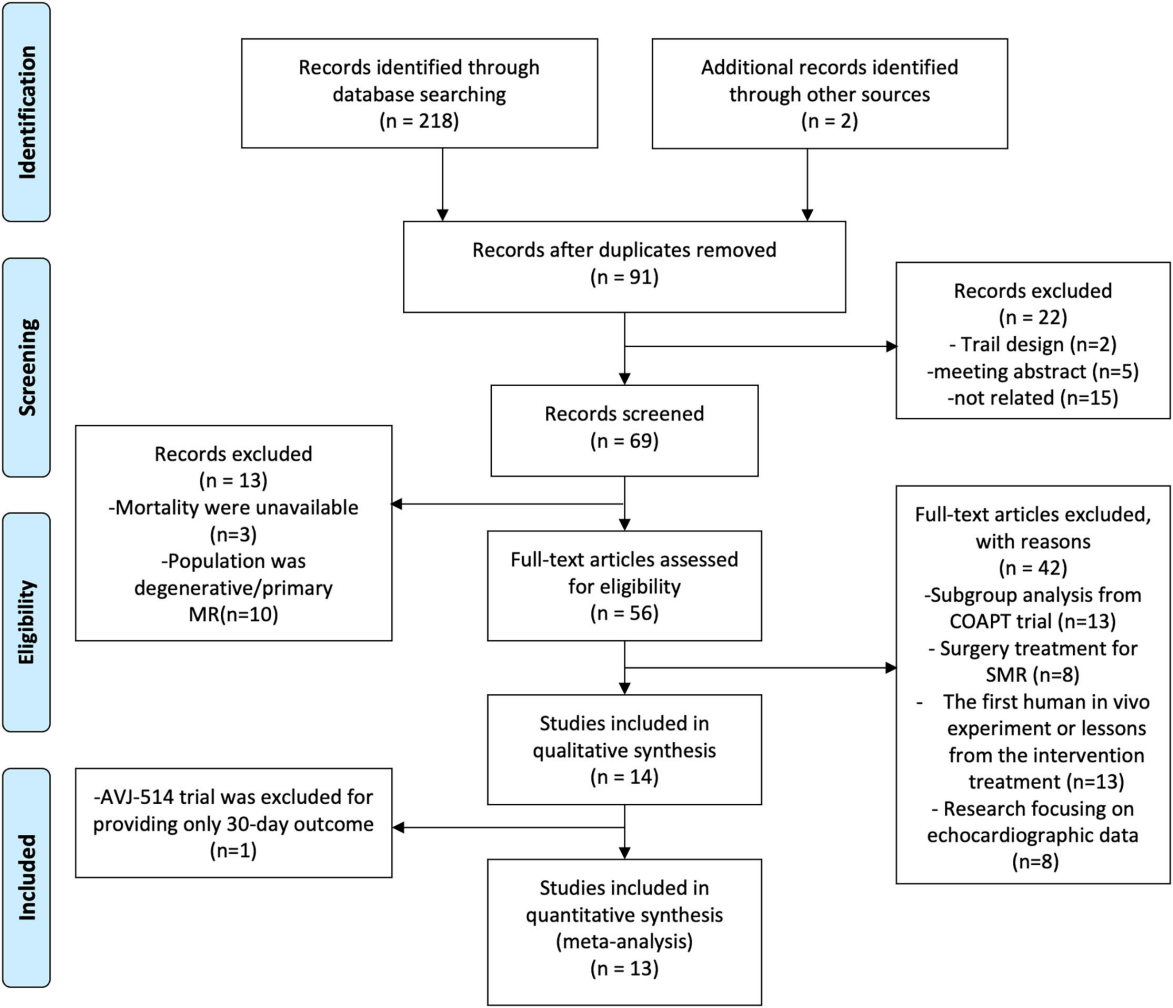
Flowchart of included studies.

Clinical research evaluating the safety and efficacy of PMVR for patients with SMR was considered for our meta-analysis. The inclusion criteria for our study included (1) patients who had severe SMR still suffered heart failure symptoms when optimal medical therapy was prescribed, (2) provided mortality and available baseline characteristic data, and (3) at least one transcatheter device was studied. We excluded review articles, duplicate studies or data, human *in vivo* experiments, and echocardiographic studies were not reviewed for inclusion (detailed information can be found in Figure 1). No language, publication date, or publication status restrictions were applied. References of prior systematic reviews and meta-analyses for related studies were also screened.

### Search Strategy and Information Sources

We used keywords related to *secondary or functional mitral regurgitation, transcatheter mitral valve repair, MitraClip*, and *mitral annuloplasty device* to search PubMed, EMBASE, and Cochrane databases through the final search date of 30 December 2021 (detailed information for search strategy can be found in Supplementary Material 2).

Two reviewers performed a systematic review, and disagreements were resolved in a panel discussion by 3 reviewers. Study selection involved screening of titles and abstracts followed by a full-text evaluation of possible eligible studies.

### Assessment of the Risk of Bias

Two independent reviewers performed the qualitative assessment and bias (low, intermediate, or high) using the Cochrane Collaboration tool (in Supplementary Material 3). Given that part of the enrolled studies were single-arm designs, the risk of publication bias was not assessed.

### Endpoint and Data Collection Process

Mortality was the only endpoint in our meta-analysis. Two reviewers independently extracted data on the study design and PMVR device (Table 1), and baseline characteristics are summarized in Supplementary Material 4. Any discrepancies between the 2 reviewers were resolved through discussion.

**Table 1:** Information of included studies.

**Study**	**Year**	**Clinical trial number**	**Study design**	**Device**	**Follow-up**
CLASP (11)	2021	NCT03170349	Multicenter, multinational, prospective, single-arm study	PASCAL repair system	2-Year
MAVERIC (12)	2021	NCT03311295	International multicenter, prospective, single arm	ARTO system	2-Year
COAPT (6)	2021	NCT01626079	Randomized, parallel-controlled, open-label multicenter trial	MitraClip device	3-Year
MITRA-FR (8)	2019	NCT01920698	Randomized, open-label multicenter trial	MitraClip device	2-Year
REDUCE FMR (13)	2019	NCT02325830	Blinded, randomized, proof-of-concept, sham-controlled trial	Carillon mitral contour system	1-Year
Messika-Zeitou et al. (14)	2018	NCT01841554	Single-arm, prospective multicentre trial	Cardioband mitral system	1-Year
Giannini et al. (15)	2016		Propensity-matched cohort trial	MitraClip	3-Year
Asgar et al. (16)	2016		Two phases, propensity matched observational study	MitraClip	1-Year
Armeni et al. (17)	2016		Retrospective, nonrandomized, propensity matched observational study	MitraClip	1-Year
Nickenig et al. (18)	2016	NCT01841554	Single-arm, multicenter, prospective trial	Cardioband system	6-Month
PTOLEMY-2 (19)	2013	NCT00787293	Prospective multicenter phase I single-arm feasibility trial	Second-generation permanent percutaneous transvenous mitral annuloplasty (PTMA) device	1-Year
TITAN (20)	2012		Prospective, non-randomized, non-blinded, multicenter trial	Carillon Mitral Contour System	1-Year
EVOLUTION (21)	2011		Multicenter, phase I single-arm trial	MONARC device	1-Year

### Statistical Analysis

We directly performed a binary random-effects proportional meta-analysis to obtain the pooled estimates of mortality and 95% confidence intervals (CIs) of included studies. Statistical heterogeneity among studies was examined using the Cochran Q statistic and the I2 statistic, with I2 being considered substantial when it was >50% (22). Leave-one-out sensitivity analysis was conducted to evaluate the key studies with substantial influence on heterogeneity (23). Meta-regression analysis was carried out to assess the potential influence of important covariates on between-study heterogeneity (significance at *P* ≤ 0.05), (24) and these models were applied to clarify whether the coexistence of these covariates explained the variability in effect estimates across all included studies for mortality. We also performed an additional sensitivity analysis to demonstrate the difference between edge-to-edge mitral valve repair devices and mitral annuloplasty devices. All data analyses were performed using statistical software. OpenMetaAnalyst (version 10.12) and RevMan (version 5.4) were used.

## Results

### Characteristics of Included Studies

Finally, 1,259 patients with SMR from 13 studies, including 3 randomized trials [COAPT (6), MITRA-FR (8), and REDUCE FMR (13)], 6 single-arm studies [CLASP (11), MAVERIC (12), David Messika-Zeitou (14), Georg Nickenig (18), PTOLEMY-2 (19), and EVOLUTION (21)], and 4 prospective trials [Cristinia Giannini (15), Asgar (16), Patrizio Armeni (17), and TITAN (20)] were involved in our meta-analysis. Among these studies, edge-to-edge repair devices were analyzed in 6 studies (CLASP, COAPT, MITRA-FR, Cristinia Giannini, Asgar, and Patrizio Armeni), while mitral annuloplasty devices were used in the other 7 studies. Detailed information on the characteristics of included studies is shown in Table 1.

### Baseline Characteristics of Included Cohorts

Demographic data, functional characteristics, history, echocardiography parameters, and medication history are summarized in Supplementary Material 4. The mean age of the enrolled population was 71.02, and 68.45% of them were men. As to the pathogenesis of SMR, ischemic diseases accounted for 59.37%.

### Pooled Mortality

**Figure 2:**
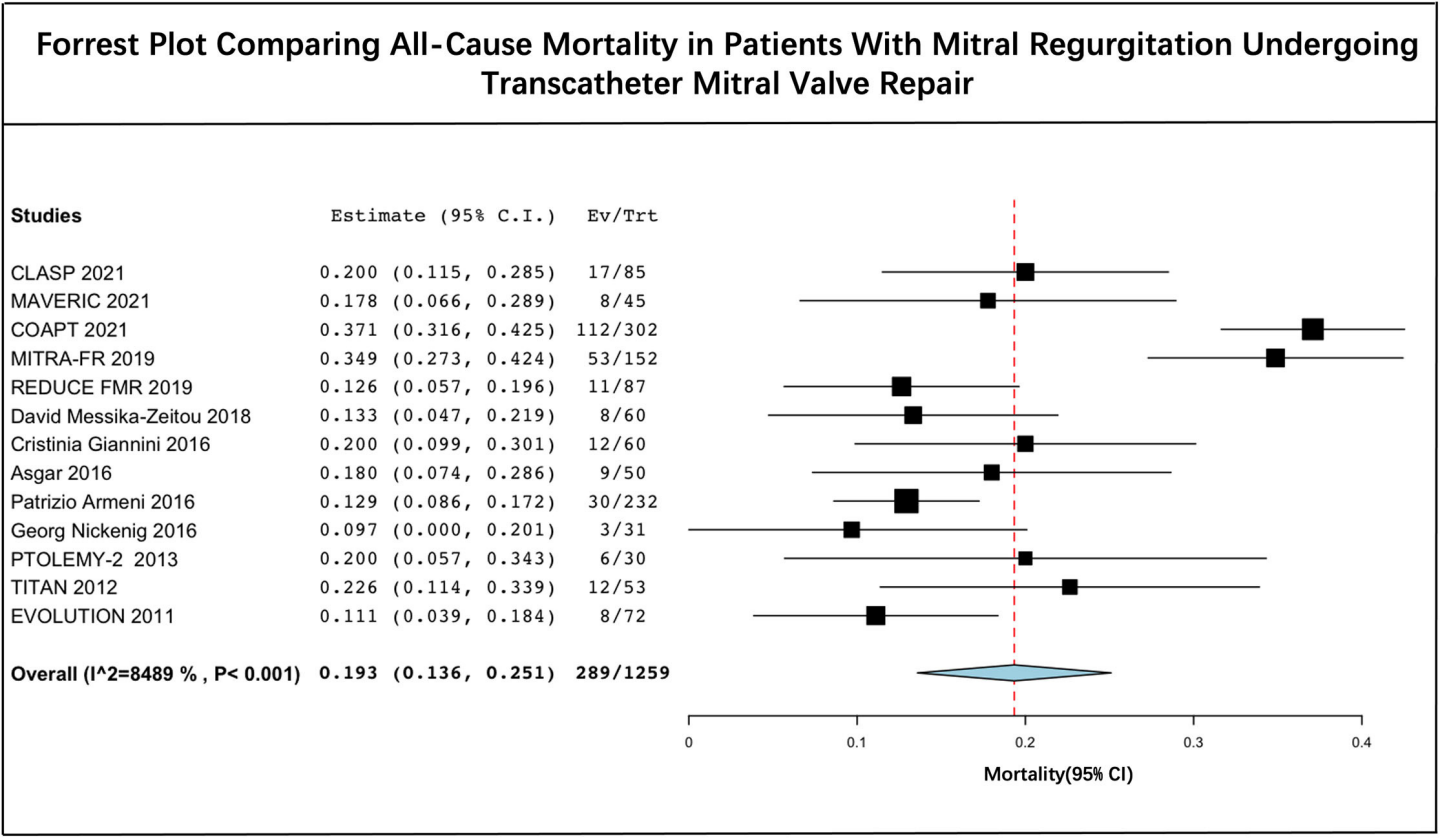
Forrest plot comparing all-cause mortality in patients with mitral regurgitation undergoing transcatheter mitral valve repair.

**Figure 3:**
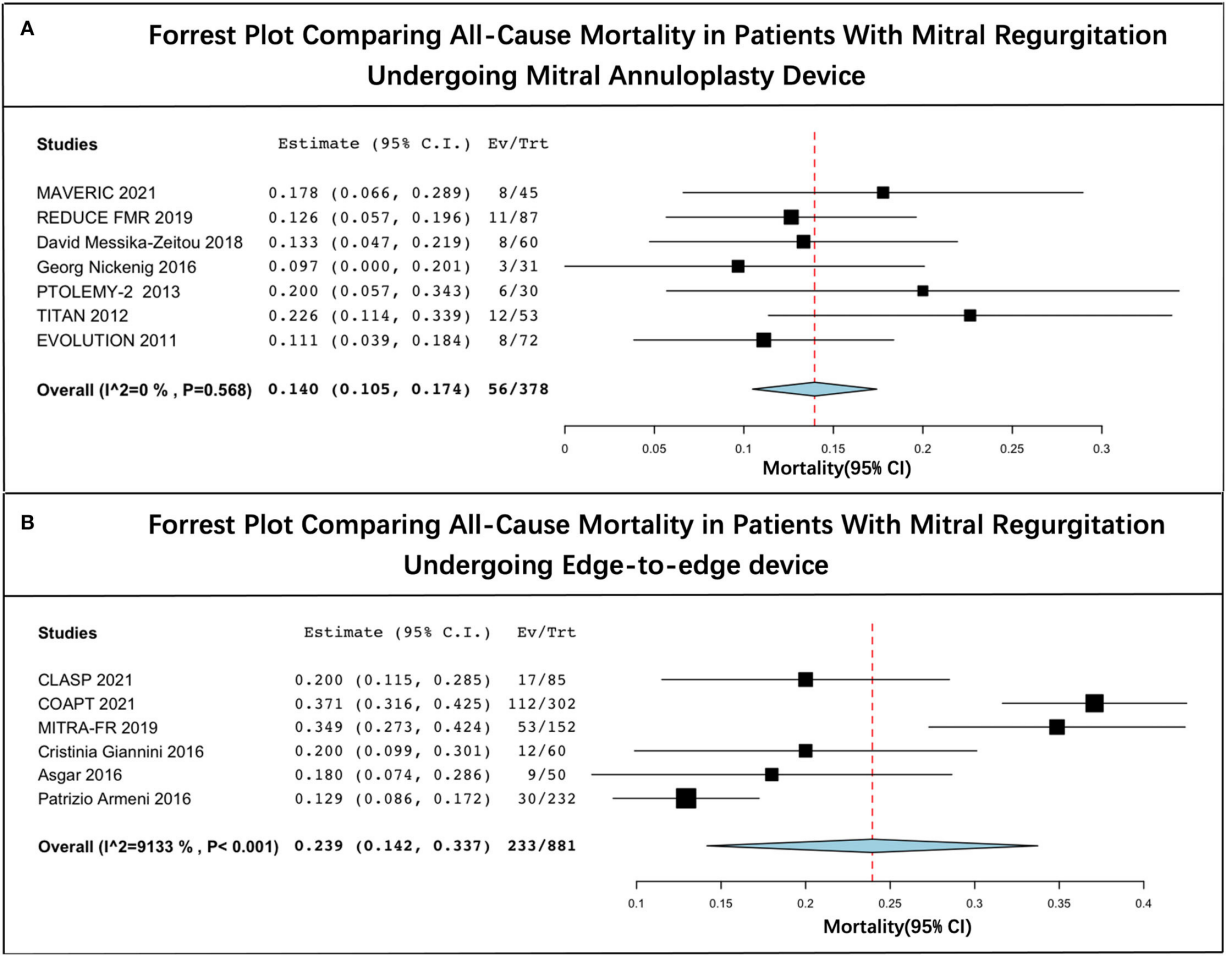
Forrest plot of subgroup analysis. **(A)** Forrest plot comparing all-cause mortality in patients with mitral regurgitation undergoing mitral annuloplasty device and **(B)** forrest plot comparing all-cause mortality in patients with mitral regurgitation undergoing edge-to-edge device.

The long-term estimated pooled mortality of PMVR was 19.3% (95% CI: 13.6–25.1) (Figure 2). A subgroup analysis was conducted to obtain the mortality associated with edge-to-edge mitral valve repair devices (23.9% [95% CI: 14.2–33.7]) and mitral annuloplasty devices (14.0% [95% CI: 10.5–17.4]), respectively (Figure 3). Leave-one-out sensitivity analysis is shown in Supplementary Material 5.

### Meta-Regression Analysis

**Figure 4:**
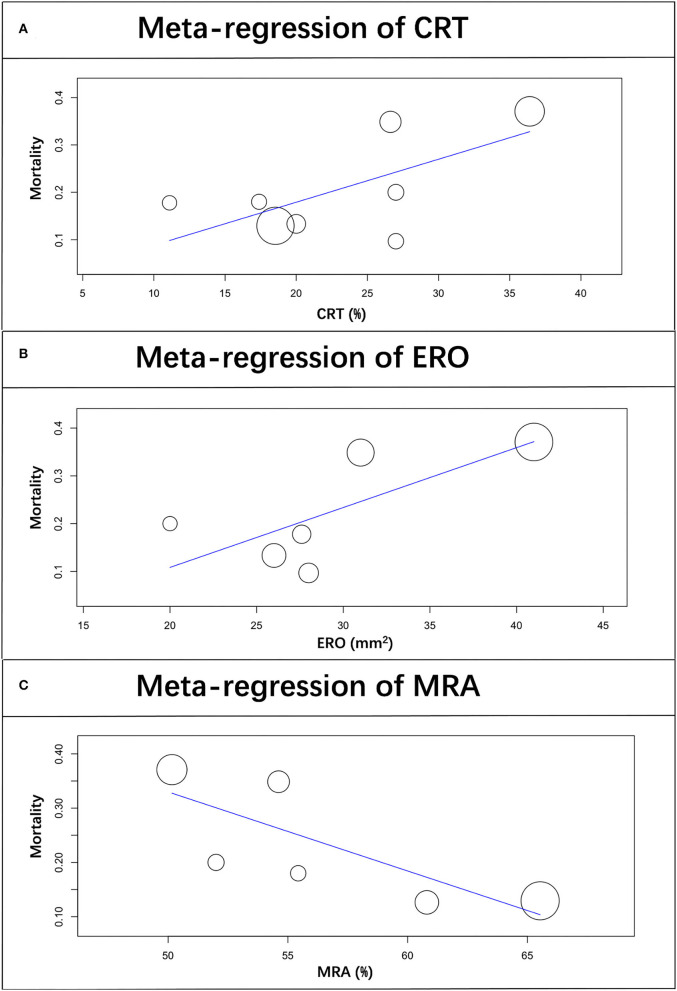
Scatterplot showing the relationship between mortality and CRT therapy **(A)**, ERO **(B)**, and MRA prescription **(C)** in patients with SMR undergoing PMVR. The size of each point correlates with the number of patients in each included study.

A meta-regression analysis was performed to assess the impact of potential baseline characteristics on pooled mortality. As shown in Table 2 and Figure 4, mortality is directly proportional to CRT therapy, ERO, and MRA use. There was a significant increase in mortality in patients with CRT therapy (β = 0.009; 95% CI: 0.002–0.016; *p* = 0.009) and larger ERO (β = 0.009; 95% CI: 0.000–0.018; *p* = 0.047), while a decrease in patients prescribed MRA (β = −0.015; 95% CI: −0.023– −0.006; *p* < 0.001).

**Table 2:** Meta-regression analysis for all-cause mortality in all patients.

**Variable**	**No. of estimates**	**Univariate**			**Multivariate**
		**β Coefficient (95% CI)**	***P*-value**	**Figure**	
CRT (%)	8/13	0.009 (0.002–0.016)	0.009	Figure 4A	NE
ERO (mm^2^)	7/13	0.009 (0.000–0.018)	0.047	Figure 4B	NE
MRA (%)	6/13	−0.015 (−0.023– −0.006)	<0.001	Figure 4C	NE

*CRT, cardiac resynchronization therapy; ERO, effective regurgitant orifice area; MRA, mineralocorticoid receptor antagonist; NE, not entered into multivariate meta-regression analysis*.

The subgroup analysis indicated that patients with preexisting AF (β = −0.002; 95% CI: −0.005– −0.000; *p* = 0.018) were associated with lower mortality if they had been subjected to a mitral annuloplasty device (Table 3A). Among those treated with an edge-to-edge repair device, the results were similar to CRT therapy and MRA. Further baseline characteristics revealing statistical significance were also identified (Table 3B).

**Table 3:** Subgroup meta-regression analysis for all-cause mortality in mitral annuloplasty device **(A)** and edge-to-edge repair device **(B)**.

**Variable**	**No. of estimates**	**Univariate**			**Multivariate**
		**β Coefficient (95% CI)**	***P*-value**	**Figure**	
**(A): Results for mitral annuloplasty device**
AF (%)	5/7	−0.002 (−0.005 to −0.000)	0.018	Supplementary Material 6A	NE
**(B): Results for edge-to-edge repair device**
CRT (%)	5/6	0.013 (0.007–0.018)	<0.001	Supplementary Material 6B	NE
MRA (%)	5/6	−0.013 (−0.022– −0.005)	0.003	Supplementary Material 6C	NE
BMI(kg/m2)	3/6	0.114 (0.042–0.186)	0.002	Supplementary Material 6D	NE
LVEF (%)	6/6	−0.050 (−0.101– −0.001)	0.046	Supplementary Material 6E	NE
LVESD (cm)	3/6	0.457 (0.229–0.686)	<0.001	Supplementary Material 6F	NE
LVESV (ml)	3/6	0.007 (0.003–0.010)	<0.001	Supplementary Material 6G	NE
LVEDV(ml)	3/6	0.013 (0.007–0.020)	<0.001	Supplementary Material 6H	NE

*AF, atrial fibrillation; CRT, cardiac resynchronization therapy; MRA, mineralocorticoid receptor antagonist; BMI, body mass index; LVESD, LV end-systolic diameter; LVESV, LV end-systolic volume; LVEDV, LV end-diastolic volume; NE, not entered into multivariate meta-regression analysis*.

## Discussion

Former meta-analyses demonstrated that, with regard to optimal medical treatment, PMVR is likely to be an efficacious and safe option (25, 26). Recently, new concepts and devices have, however, provided disparate evidence on this topic, rendering it necessary to reevaluate the effect of PMVR.

To the best of our knowledge, this is the largest and most advanced meta-analysis to date. Follow-up mortality among patients with SMR after PMVR has been evaluated, and the estimated pooled mortality is 19.3%, with a 95% CI ranging from 13.0 to 25.5%. However, the I^2^ statistic showed significant heterogeneity among studies. Further leave-one-out analysis and subgroup analysis indicated that the COAPT and MITRA-FR trials were the main sources of heterogeneity. COAPT and MITRA-FR trials were the first two randomized trials to evaluate the efficacy of PMVR in symptomatic patients with severe secondary mitral regurgitation using optimal medical therapy in accordance with guidelines. Their contradictory conclusions generated additional thought with regard to the selection of specific patients who could benefit the most from PMVR. We, therefore, conducted this meta-analysis to detect potential clinical factors associated with mortality by meta-regression.

In the past, EROA has been recognized as a strong predictor of mortality in mitral regurgitation (27). PMVR changes this conclusion. An echocardiographic analysis (28) from a COAPT trial indicated that greater ERO led to adverse outcomes during follow-up, as observed in optimal medical therapy patients, and showed no significant prognostic value among patients undergoing PMVR. Similarly, Nicole's research (29) affirmed that groups with different baseline EROs exhibited relevant clinical improvements after TMVR. We found, however, that ERO was positively associated with mortality after PMVR, a finding which contradicts the results of individual studies. A meta-regression is an efficient way of detecting potential variates with heterogeneity. Unfortunately, we failed to perform multivariate regression analyses for a limited number of baseline characteristics. This may have affected our conclusions as a result of unadjusted bias. Further research should, therefore, be conducted to clarify the association between ERO and mortality after PMVR.

Each article claims that all enrolled patients had undergone the optimal medical therapy. We noticed that guideline-recommended drugs for heart failure (30) were not fully prescribed. Hyperkalaemia is a major concern for cessation of MRA and our meta-regression analysis indicated that MRA was associated with better outcomes. Patiromer, a novel potassium binder, has been proven to improve adherence to MRA (31). It is hoped that this clinical trial increases the use of guideline-recommended drugs for heart failure.

Although subgroup analysis of a COAPT trial (32) suggested that MitraClip could decrease the 2-year mortality rate, regardless of prior CRT implantations, our meta-regression results from pooled data showed that CRT might be associated with poor prognosis. CRT and maximally tolerated guideline-directed medical therapy were preferential to PMVR, and CRT was especially recommended in patients with LVEF ≤ 35% (4, 30). However, HF symptoms and moderate-severe or severe SMRs persist or worsen in 30–40% of patients after CRT implantation, which means a poor long-term prognosis (33). This indicates that among patients with worsening cardiac functions, rigid adherence to guidelines may lead them to miss the optimal timing of PMVR, resulting in a reduced benefit. Therefore, further research is necessary to prove that PMVR should be prioritized over CRT, especially in patients with better cardiac function.

Subgroup analysis according to different treatment strategies provides us with a perspective to better understand the underlying factors affecting prognosis and the mechanism of SMR. The concept of atrial functional MR (AFMR) is becoming well-accepted with reference to patients with AF suffering significant mitral regurgitation without LV systolic dysfunction (34). Although the underlying mechanism is not well-clarified, impaired mitral annulus dynamics seem to contribute more than LA remodeling (35, 36). AF is an independent negative predictor of long-term mortality among patients with MitraClip implantations (26, 37, 38). Our sub-analysis of patients who were treated with a mitral annuloplasty device demonstrated that patients with AF benefited the most. To the best of our knowledge, this finding was first reported in our study. This also emphasizes that the pathogenesis of AFMR is related to the mitral annulus, and ring annuloplasty can improve the prognosis of these patients (39).

We identified LVEF as a positive variate associated with lower mortality among patients who had undergone the use of an edge-to-edge repair device. LVESD, LVESV, and LVEDV were identified as negative variates, suggesting that patients with poor cardiac function might not benefit from PMVR. A former meta-analysis (40) of RCTs and propensity score-matched observation studies to assess the role of percutaneous mitral valve repair also indicated that a patient with a greater LVEDV at baseline was less likely to benefit from PMVR. Moreover, a *post-hoc* analysis from a COAPT trial also revealed that a higher baseline NYHA functional class was strongly associated with a greater risk for adverse events (41). Therefore, valve intervention is generally not recommended as an option when LVEF is <15% (30) in order to decrease the possibility of the reverse of LV remodeling.

## Limitations

There are some limitations requiring attention. First, only 3 randomized data were enrolled, and the limitations of a retrospective study design could not be avoided. Second, because of the limited number of studies reporting the vital baseline characteristics, we were unable to conduct a meta-regression for these variates. We also failed to perform a multivariate regression analysis for the same reason, and confounding bias for the conclusion cannot be ignored. Third, although we discarded the data from the control group in controlled trials, data from single-arm designs may have resulted in bias in our meta-analysis. Nevertheless, meta-regression analysis is helpful in understanding heterogeneity and providing a perspective according to which patients may be stratified in terms of who may be best able to benefit from PMVR.

## Data Availability Statement

The original contributions presented in the study are included in the article/Supplementary Material, further inquiries can be directed to the corresponding author.

## Author Contributions

WS: drafting of the manuscript, had full access to all of the data in the study, takes responsibility for the integrity of the data, and the accuracy of the data analysis. WS and CD: concept and design, statistical analysis, administrative, and technical or material support. WZ, DZ, and GY: acquisition and analysis or interpretation of data. WZ and CD: supervision. All authors: critical revision of the manuscript for important intellectual content.

## Conflict of Interest

The authors declare that the research was conducted in the absence of any commercial or financial relationships that could be construed as a potential conflict of interest.

## Publisher's Note

All claims expressed in this article are solely those of the authors and do not necessarily represent those of their affiliated organizations, or those of the publisher, the editors and the reviewers. Any product that may be evaluated in this article, or claim that may be made by its manufacturer, is not guaranteed or endorsed by the publisher.
