# Supplementary material for: Mortality and Clinical Predictors After Percutaneous Mitral Valve Repair for Secondary Mitral Regurgitation: A Systematic Review and Meta-Regression Analysis
Source: Front Cardiovasc Med. 2022 Jul 4;9:918712. doi: 10.3389/fcvm.2022.918712 (PMC9289259; doi:10.3389/fcvm.2022.918712)

Supplementary Online Content

Supplement 1: PRISMA checklist
Supplement 2: Search Strategy
Supplement 3: Quality Assessment and Publication Bias
Supplement 4: Baseline Characteristics
Supplement 5: Leave-one-out Meta-analysis
Supplement 6: Subgroup Meta-regression Analysis for Mortality

This supplementary material has been provided by the authors to give readers additional information about their work.

Supplement 1 PRISM Checklist

Section/topic	#	Checklist item	Reported on page #
**TITLE**			
Title	1	Identify the report as a systematic review, meta-analysis, or both.	1
**ABSTRACT**			
Structured summary	2	Provide a structured summary including, as applicable: background; objectives; data sources; study eligibility criteria, participants, and interventions; study appraisal and synthesis methods; results; limitations; conclusions and implications of key findings; systematic review registration number.	2
**INTRODUCTION**			
Rationale	3	Describe the rationale for the review in the context of what is already known.	3
Objectives	4	Provide an explicit statement of questions being addressed with reference to participants, interventions, comparisons, outcomes, and study design (PICOS).	3
**METHODS**			
Protocol and registration	5	Indicate if a review protocol exists, if and where it can be accessed (e.g., Web address), and, if available, provide registration information including registration number.	3
Eligibility criteria	6	Specify study characteristics (e.g., PICOS, length of follow-up) and report characteristics (e.g., years considered, language, publication status) used as criteria for eligibility, giving rationale.	3
Information sources	7	Describe all information sources (e.g., databases with dates of coverage, contact with study authors to identify additional studies) in the search and date last searched.	3-4
Search	8	Present full electronic search strategy for at least one database, including any limits used, such that it could be repeated.	4

Study selection	9	State the process for selecting studies (i.e., screening, eligibility, included in systematic review, and, if applicable, included in the meta-analysis).	4
Data collection process	10	Describe method of data extraction from reports (e.g., piloted forms, independently, in duplicate) and any processes for obtaining and confirming data from investigators.	4
Data items	11	List and define all variables for which data were sought (e.g., PICOS, funding sources) and any assumptions and simplifications made.	4
Risk of bias in individual studies	12	Describe methods used for assessing risk of bias of individual studies (including specification of whether this was done at the study or outcome level), and how this information is to be used in any data synthesis.	4
Summary measures	13	State the principal summary measures (e.g., risk ratio, difference in means).	4
Synthesis of results	14	Describe the methods of handling data and combining results of studies, if done, including measures of consistency (e.g., I^2) for each meta-analysis.	4

Section/topic	#	Checklist item	Reported on page #
Risk of bias across studies	15	Specify any assessment of risk of bias that may affect the cumulative evidence (e.g., publication bias, selective reporting within studies).	4
Additional analyses	16	Describe methods of additional analyses (e.g., sensitivity or subgroup analyses, meta-regression), if done, indicating which were pre-specified.	4
**RESULTS**			
Study selection	17	Give numbers of studies screened, assessed for eligibility, and included in the review, with reasons for exclusions at each stage, ideally with a flow diagram.	4-5

Study characteristics	18	For each study, present characteristics for which data were extracted (e.g., study size, PICOS, follow-up period) and provide the citations.	5
Risk of bias within studies	19	Present data on risk of bias of each study and, if available, any outcome level assessment (see item 12).	5
Results of individual studies	20	For all outcomes considered (benefits or harms), present, for each study: (a) simple summary data for each intervention group (b) effect estimates and confidence intervals, ideally with a forest plot.	5
Synthesis of results	21	Present results of each meta-analysis done, including confidence intervals and measures of consistency.	6
Risk of bias across studies	22	Present results of any assessment of risk of bias across studies (see Item 15).	4
Additional analysis	23	Give results of additional analyses, if done (e.g., sensitivity or subgroup analyses, meta-regression [see Item 16]).	7
**DISCUSSION**			
Summary of evidence	24	Summarize the main findings including the strength of evidence for each main outcome; consider their relevance to key groups (e.g., healthcare providers, users, and policy makers).	8
Limitations	25	Discuss limitations at study and outcome level (e.g., risk of bias), and at review-level (e.g., incomplete retrieval of identified research, reporting bias).	9
Conclusions	26	Provide a general interpretation of the results in the context of other evidence, and implications for future research.	9
**FUNDING**			
Funding	27	Describe sources of funding for the systematic review and other support (e.g., supply of data); role of funders for the systematic review.	10

Supplement 2 Search Strategy

PICOS strategy was applied in our search strategy

P (patients): mitral regurgitation (MR); mitral insufficiency; secondary/functional MR (SMR/FMR)

I (intervention): transcatheter mitral valve repair (TMVR); percutaneous mitral valve intervention (PMVR); mitral valve repair; transcathether edge-to-edge repair (TEER); mitra clip; mitral annuloplasty device; Cardioband; Carillon

C (comparison): This is a single-arm meta-analysis, we did not set a control group. Therefore, no search term was applied.

O (outcome): mortality; death; all-cause death

S (studies design): clinical trial; randomized controlled trial (RCT); Observational study; cohort studies (including prospective/retrospective studies)

Supplement 3 Quality Assessment and Publication Bias

	Random sequence	Allocation concealment	Blinding of participants and Personnel *	Blinding of outcome assessment	Incomplete outcome data	Selective reporting	Other sources of bias***
CLASP /2021	🟩	🟨	🟥	🟨	🟩	🟩	🟩
MAVERIC /2021	🟩	🟨	🟥	🟨	🟩	🟩	🟩
COAPT /2021	🟩	🟩	🟩	🟩	🟩	🟩	🟩
MITRA-FR /2019	🟩	🟩	🟩	🟩	🟩	🟩	🟩
REDUCE FMR /2019	🟩	🟩	🟩	🟩	🟩	🟩	🟩
Messika-Zeitou /2018	🟩	🟩	🟥	🟨	🟩	🟩	🟩
Cristinia Giannini /2016	🟩	🟩	🟥	🟨	🟩	🟩	🟩
Asgar / 2016	🟩	🟩	🟥	🟨	🟩	🟩	🟩
Patrizio Armeni/2016	🟩	🟩	🟥	🟨	🟩	🟩	🟩
Georg Nickenig /2016	🟩	🟨	🟥	🟨	🟩	🟩	🟩
PTOLEMY-2 /2013	🟨	🟨	🟥	🟥	🟩	🟩	🟩
TITAN / 2012	🟨	🟨	🟥	🟥	🟩	🟩	🟩
EVOLUTION /2011	🟨	🟨	🟥	🟥	🟩	🟩	🟩

🟩 Low risk of bias 🟨 Unclear risk of bias 🟥 High risk of bias

* COAPT, MITRA-FR, REDUCE used an open label trial design, the participants and outcome assessment were all blinded. Others were retrospective studies which shared a high risk of bias.

Supplement 4: Baseline Characteristics

Study	Patient No	Age	Male (%)	I-SMR (%)	Device	Functional characteristics				Prior history							Echocardiography parameters						Medication history					
						6MWD	NHYA3/4 (%)	MR3+ (%)	MR4+ (%)	DM(%)	HP(%)	MI(%)	Stroke(%)	COPD (%)	AF(%)	CKD (%)	LVEF	LVESD	LVEDD	LVESV	LVEDV	EROA	CRT	Pacemaker/AICD	Beta blockers	ACEi/ ARB/ ARNi	MRA	Diuretic agent
CLASP	85	72.2	55.3	41.9	PASCAL repair system	278.0	51	58	42	29.0	66.9	36.3	15.3	5.6	53.4	24.2	43.8	49.5	61.8	108.7	181.2	38.0						
MAVERIC	45	69.8	60.0	37.8	ARTO system	280.0	73.4			20.0	46.7		6.7	20.0	44.4	51.0	40.2	54.0	63.0			27.6	11.1	26.7	88.9	84.4		97.7
COAPT	302	72.3	64.0	60.8	MitraClip device	254.6	60.8	52.1	47.7	37.3	80.5	51.5	17.1	23.3	55.2	71.8	31.3	53.0	62.0	134.9	194.4	41.0	36.4	31.3	90.4	67.1	50.16	89.1
MITRA-FR	152	70.4	74.7	59.2	MitraClip device		67.1	37.0	61.0	29.3		41.8			31.9	13.5	33.1					31.0	26.6	34.5	89.5	84.0	54.6	98.7
REDUCE FMR	87	70.1	63	66.7	Carillon mitral contour system	306.4	53.2	28.8	5.9	24.0					51.0		34.0	55.0	64.0	126.1	187.4				90.8	90.0	60.8	98.3
David	60	69.8	72.5	60.0	Cardioband mitral system	302.6	87.0	27.0	73.0	30.0					59.2		33.0					26.0	20.0	33.0				
Cristinia Giannini	60	75.0	67.0	52	MitraClip		75.0	49.0	46.0	29.0	59.0	38.0	0.8	23.0	39.0	41.0	34.0	49.0	64.0	113.0	182.0		27.0	8.0	68.0	64.0	52.0	90.0
Asgar	50	72.1	76.1	75	MitraClip		63.4	66.3	33.7	40.0	57.6				60.9		35.3						17.4	45.7	84.8	70.7	55.4	87.0
Patrizio Armeni	232	71.0	73.9	60.1	MitraClip					30.0	93.6	45.0	8.1	23.2	33.2		33.2						18.5	28.2	74.7	80.7	65.5	
Georg Nickenig	31	71.8	83.9	61.0	Cardioband system	250.0	97.0			45.0	65.0				77.0	77.0	34.0					28.0	27.0	32.0				
PTOLEMY-2	30	72	63.0	57.0	second-generation (PTMA) device		67.0			10.0	53.0	37.0	3.0	7.0	27.0	27.0	38.0			117.0	175.0	20.0						
TITAN	53	62.44	77.4	64.2	Carillon Mitral Contour System	314.0		56.6	26.4	20.8							28.1	58.0	67.0	217.8	160.1			15.1				
EVOLUTION	72	70	82.0	68.0	MONARC device		54.0	21.0	17.0	24.0	54.0	57,0					37.8											

I-SMR, Ischaemic second mitral regurgitation; 6MWD, 6-min walk distance; NHYA, New York Heart Association; MR, mitral regurgitation; DM; HP, hypertension; MI, myocardial infarction; COPD, chronic obstructive pulmonary disease; AF, atrial fibirillation; CKD, chronic kidney disease; LVEF, left ventricular ejection fraction; LVESD, left ventricular end-systolic diameter; LVEDD, left ventricular end-diastolic diameter; LVESV, left ventricular end-systolic volume; LVEDV, left ventricular end-diastolic volume; ERO, effective regurgitant orifice area; CRT, cardiac resynchronization therapy; AICD, automatic implantable cardioverter-defibrillator; ACEi, angiotensin-converting enzyme inhibitors; ARB, angiotensin II receptor blocker; ARNi, angiotensin II receptor blocker neprilysin inhibitor; MRA, mineralocorticoid receptor antagonist

Supplement 5: Leave-one-out Meta-analysis

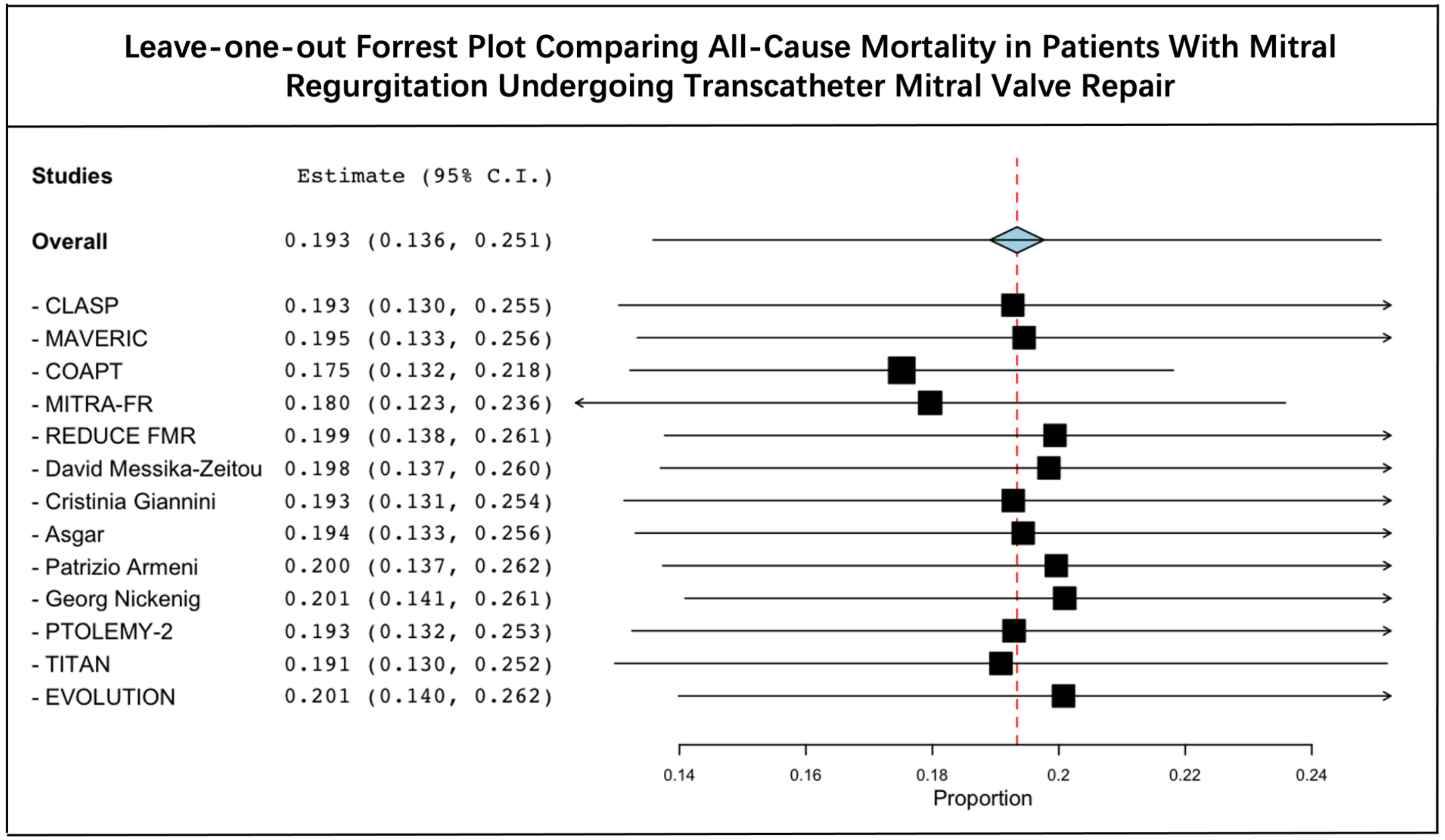

Supplement 6: Subgroup Meta-regression Analysis for Mortality

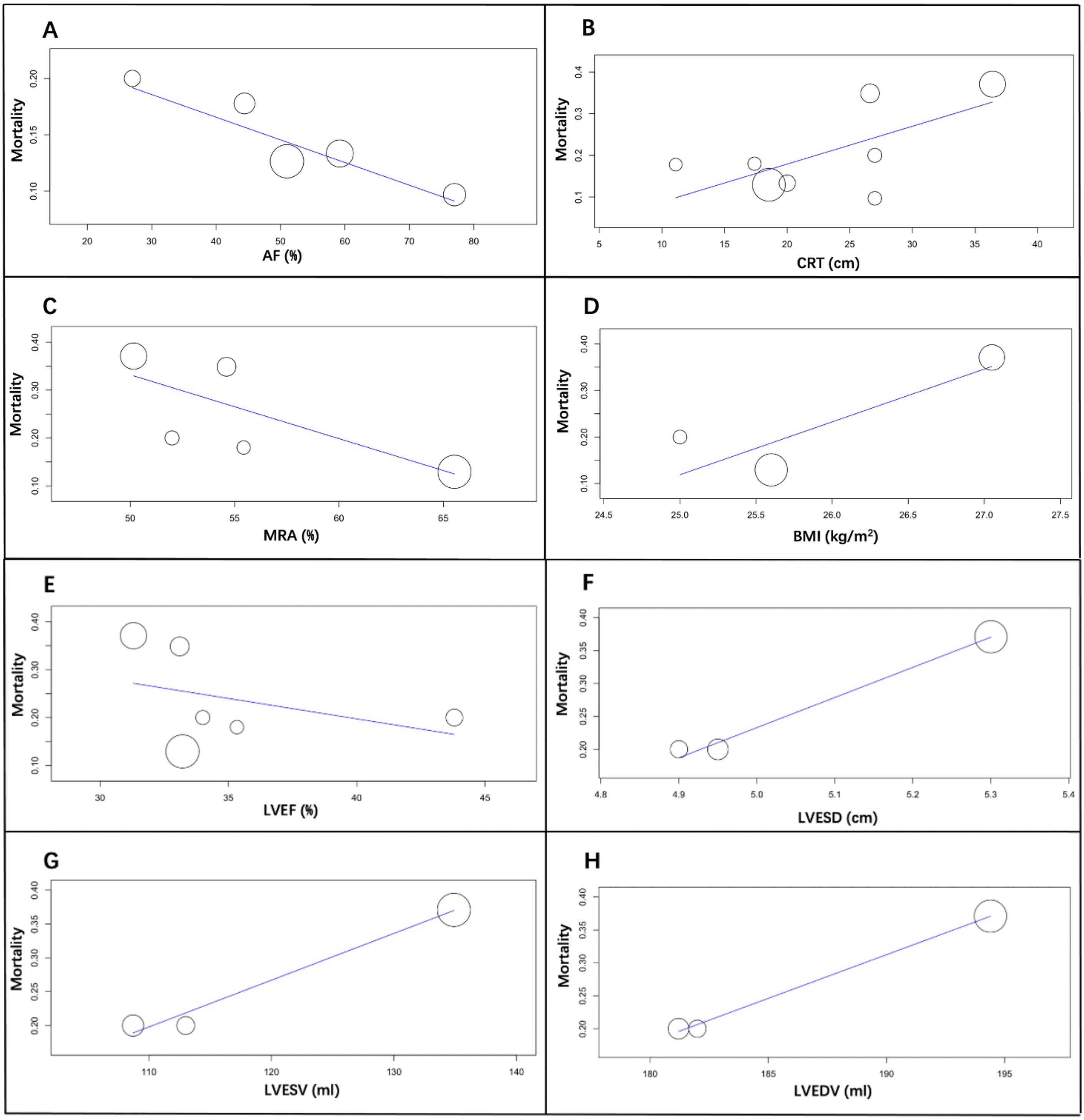

Supplement: Supplementary file 1 [file Presentation_1.pdf]
